# A mathematical model of the euglycemic hyperinsulinemic clamp

**DOI:** 10.1186/1742-4682-2-44

**Published:** 2005-11-03

**Authors:** Umberto Picchini, Andrea De Gaetano, Simona Panunzi, Susanne Ditlevsen, Geltrude Mingrone

**Affiliations:** 1 CNR-IASI BioMatLab, Rome, Italy; 2 Department of Biostatistics, University of Copenhagen, Denmark; 3 Istituto di Medicina Interna e Geriatria, Divisione di Malattie del Ricambio, Università Cattolica del Sacro Cuore, Policlinico Universitario "A. Gemelli", Rome, Italy

## Abstract

**Background:**

The Euglycemic Hyperinsulinemic Clamp (EHC) is the most widely used experimental procedure for the determination of insulin sensitivity, and in its usual form the patient is followed under insulinization for two hours. In the present study, sixteen subjects with BMI between 18.5 and 63.6 kg/m^2 ^were studied by long-duration (five hours) EHC.

**Results:**

From the results of this series and from similar reports in the literature it is clear that, in obese subjects, glucose uptake rates continue to increase if the clamp procedure is prolonged beyond the customary 2 hours. A mathematical model of the EHC, incorporating delays, was fitted to the recorded data, and the insulin resistance behaviour of obese subjects was assessed analytically. Obese subjects had significantly less effective suppression of hepatic glucose output and higher pancreatic insulin secretion than lean subjects. Tissue insulin resistance appeared to be higher in the obese group, but this difference did not reach statistical significance.

**Conclusion:**

The use of a mathematical model allows a greater amount of information to be recovered from clamp data, making it easier to understand the components of insulin resistance in obese vs. normal subjects.

## Background

With the growing epidemiological importance of insulin resistance states such as obesity and Type 2 Diabetes Mellitus, T2DM, and with increasing clinical recognition of the impact of the so-called metabolic syndrome, the assessment of insulin sensitivity has become highly relevant to metabolic research.

The experimental procedures currently employed to gather information on the degree of insulin resistance of a subject are the Oral Glucose Tolerance Test (OGTT), the Intra-Venous Glucose Tolerance Test (IVGTT), the Euglycemic Hyperinsulinemic Clamp (EHC), the Hyperglycemic Clamp, the insulin-induced hypoglycemia test (*K*_*ITT*_), and less commonly used methods based on tracer administration [1-3]. Of these, the EHC is considered the tool of choice in the diabetological community, in spite of its labor-intensive execution, because it is usually considered that the results obtained can be interpreted simply [4,5]. The favor with which the EHC is viewed in this context stems in part from the belief that while mathematical models of the glucose insulin system make untenable assumptions, the EHC approach is relatively assumption-free, or model-independent.

In general, insulin resistance expresses an imbalance between the amount of pancreatic insulin secreted in response to a glucose load and the levels of plasma glucose attained. In other words, in order to obtain the same plasma glucose concentration, higher levels of plasma insulin are necessary in insulin-resistant subjects than in normal controls [6].

The clamp, as usually employed, yields easy-to-compute indices, which are commonly used as measures of insulin resistance. The M value [5] is defined as the average glucose infusion rate over a period of 80–120 minutes from the start of the insulin infusion. The M/I ratio is the ratio of the M value to the average plasma insulin concentration during the same period. If a two-step clamp is performed (though see negative comments [4]) the ΔM/ΔI ratio is defined as the increment of M produced by raising the insulin infusion rate over the corresponding increment of I. The use of these indices, however, makes two fundamental assumptions: first, that at the end of 120' of insulin infusion the experimental subject is at steady state with regard to glucose uptake rate; and second, that the glucose uptake rate increases linearly with increasing insulinemia, either throughout the insulin concentration range (when using the M/I index for characterizing the subject's response) or between successive insulin concentrations reached in the two-step clamp (when using the ΔM/ΔI index). These assumptions are, however, only a first approximation to the real state of things. On the one hand, it has already been shown that if a clamp experiment is continued beyond the customary 2 hours " [...] glucose utilization increases progressively through(out) five hours of moderate hyperinsulinemia." [7]. On the other hand [8], carefully measured average glucose uptake rates at two hours are nonlinearly related to increasing levels of plasma insulin, and from the reported data, glucose uptake may approach a maximal value asymptotically as insulinemia increases. In spite of these observations, the vast majority of experimental diabetologists ([9], [4], [10]) consider the EHC the procedure of choice and many studies have already been conducted using it. It would be interesting to be able to reinterpret this vast mass of observations using a more explicitly quantitative approach. The goal of the present work is to formulate a model of the EHC and fit it to EHC data recorded from human subjects. The structure of the model we have developed allows us to discuss the mechanisms whereby a sufficiently long insulin infusion might be able to increase glucose uptake progressively, and to explore the possible implications of the commonly observed insulin resistance pattern in obese subjects.

## Methods

### Subjects

Sixteen subjects were enrolled in the study, 8 normal volunteers and 8 patients from the Obesity Outpatient Clinic of the Department of Internal Medicine at the Catholic University School of Medicine. For one normal subject the recorded glycemia values were accidentally lost and this subject was therefore discarded from the following mathematical analysis. The subjects had widely differing BMIs (from 18.5 to 63.6). All subjects were clinically euthyroid, had no evidence of diabetes mellitus, hyperlipidemia, or renal, cardiac or hepatic dysfunction and were undergoing no drug treatments that could have affected carbohydrate or insulin metabolism. The subjects consumed a weight-maintaining diet consisting of at least 250 g of carbohydrate per day for 1 week before the study. Table 1 reports the main anthropometric and metabolic characteristics of the subjects.

**Table 1:** Anthropometric and metabolic characteristics for lean (BMI ≤ 25) and overweight or obese (BMI > 25) subjects.

	**Lean subjects (n = 7)**	**Overweight and Obese subjects (n = 8)**	**p**
BMI [kg/m^2^]	20.0 [18.5, 22.7]	37.0 [27.8, 63.6]	**0.001**
BSA [m^2^]	1.55 [1.49, 1.73]	2.1 [1.83, 2.38]	**0.001**
G_fast _[mM]	3.67 [3.4, 5.4]	5.2 [4.61, 5.9]	**0.024**
I_fast _[pM]	27.8 [13.9, 49.4]	123.7 [79.2, 152.9]	**0.001**
I_max _[pM]	482.14 [464.5, 526.9]	606.3 [497.3, 683.2]	**0.004**

Values are median [min, max]. All comparisons were performed by the Mann-Whitney U-test. BSA is the Body Surface Area [m^2^] calculated via the DuBois formula (BSA = 0.20247 · height ^0.725 ^[m] · weight ^0.425 ^[kg])

The study protocol followed the guidelines of the Medical Ethics Committee of the Catholic University of Rome Medical School; written informed consent was obtained from all subjects.

### Experimental protocol

Each subject was studied in the postabsorptive state after a 12–14 h overnight fast. Subjects were admitted to the Department of Metabolic Diseases at the Catholic University School of Medicine in Rome the evening before the study. At 07.00 hours on the following morning, the infusion catheter was inserted into an antecubital vein; the sampling catheter was introduced in the contralateral dorsal hand vein and this hand was kept in a heated box (60°C) in order to obtain arterialized blood. A basal blood sample was obtained in which insulin and glucose levels were measured. At 08.00 hours, after a 12–14 h overnight fast, the Euglycemic Hyperinsulinemic glucose Clamp was performed according to [5]. A priming dose of short-acting human insulin was given during the initial 10 min in a logarithmically decreasing manner so that the plasma insulin was raised acutely to the desired level. During the five-hour clamp procedure, the glucose and insulin levels were monitored every 5 min and every 20 min respectively, and the rate of infusion of a 20% glucose solution was adjusted during the procedure following the published algorithm [5]. Because serum potassium levels tend to fall during this procedure, KCl was given at a rate of 15–20 mEq/h to maintain the serum potassium between 3.5 and 4.5 mEq/l.

Serum glucose was measured by the glucose oxidase method using a Beckman Glucose Analyzer II (Beckman Instruments, Fullerton, Calif., USA). Plasma insulin was measured by microparticle enzyme immunoassay (Abbott Imx, Pasadena, Calif., USA).

### Modelling

In order to explain the oscillations of glycemia occurring in response to hyperinsulinization and to continuous glucose infusion at varying speeds, we hypothesized the following system:







where

ω(s) = α^2^se^-αs^, T_gx_(s) = 0 ∀s∊ [-τ_g_,0] and T_ix_(0) = T_ixb_.

**Figure 1:**
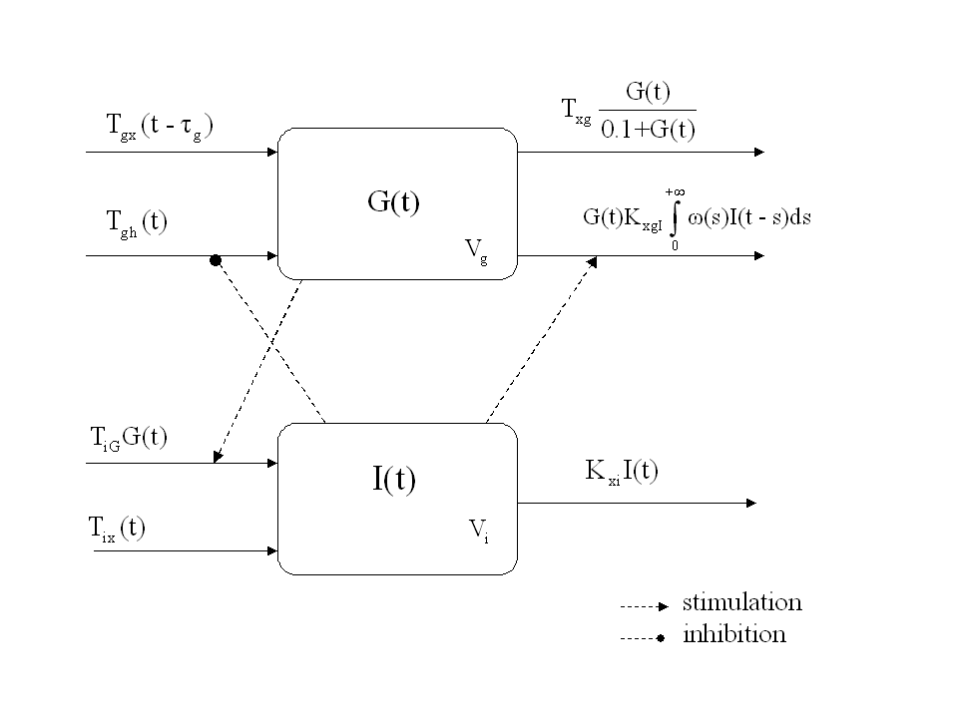
Schematic representation of the model (1), (2) and (3).

T_gx_(t) and T_ix_(t) are (input or forcing) state variables of which the values are known at each time; the state variables and the parameters are defined in tables 2 and 3. The model is diagrammatically represented in Figure 1.

**Table 2:** Definitions of the state variables.

**Variables**	
G(t) [mM]	plasma glucose concentration at time t
I(t) [pM]	serum insulin concentration at time t
t [min]	time from insulin infusion start
T_gx_(t) [mmol/min/kgBW]	glucose infusion rate at time t
T_ix_(t) [pmol/min/kgBW]	insulin infusion rate at time t
T_gh_(t) [mmol/min/kgBW]	net Hepatic Glucose Output (HGO) at time t

**Table 3:** Definitions of the parameters.

**Parameters**	
G_b _[mM]	basal glycemia
I_b _[pM]	basal insulinemia
T_xg _[mM / min]	maximal insulin-independent rate constant for glucose tissue uptake
K_xgI _[min^-1^/pM]	insulin-dependent apparent first-order rate constant for glucose tissue uptake at insulinemia I
K_xi _[min^-1^]	apparent first-order rate constant for insulin removal from plasma
T_iG _[pM/min/mM]	apparent zero-order net insulin synthesis rate at unit glycemia (after liver first-pass effect)
T_ixb _[pmol/min/kgBW]	basal insulin infusion rate, which is given by the measured value of T_ix _at time zero according to [18]
T_ghmax _[mmol/min/kgBW]	maximal Hepatic Glucose Output at zero glycemia, zero insulinemia
T_ghb _[mmol/min/kgBW]	basal value of T_gh_
V_g _[L/kgBW]	volume of distribution for glucose
V_i _[L/kgBW]	volume of distribution for insulin
*α *[#]	time constant for the insulin delay kernel ω(·)
τ_g _[min]	discrete (distributional) delay of the change in glycemia following glucose infusion
λ [mM^-1^pM^-1^]	rate constant for Hepatic Glucose Output decrease with increase of glycemia and insulinemia
ρ [#]	average delay of insulin effect

Equations (1) and (2) express the variations of plasma glucose and plasma insulin concentrations. Equation (3) represents the rate of net Hepatic Glucose Output, starting at maximal HGO at zero glucose and zero insulin and decaying monotonically with increases in both glucose and effective insulin concentrations in the plasma.

The variation of glucose concentration in its distribution space may be attributed to the external glucose infusion rate, liver glucose output and delayed-insulin-dependent as well as insulin-independent glucose tissue uptake. Infused glucose raises glycemia after a delay τ_g _due to the time required to equilibrate the intravenously infused quantity throughout the distribution space. The net HGO is assumed to be equal to T_ghb _at the beginning of the experiment and to decrease toward zero as glycemia or insulinemia levels increase. Serum insulin, after a delay depending on its transport to the periphery and the subsequent activation of cellular membrane glucose transporters, affects glucose clearance through equation (1) and the glucose synthesis rate through equation (3).

We hypothesize that ω(s) represents the density of the metabolic effect at time t for unit serum insulin concentration at time t - s (s ≤ t). We could choose ω(s) as a single function or as a linear combination of functions (with positive coefficients adding up to unity) from the family of Erlang-functions:



The first two functions of the family are

ω^(1) ^(s) = αe^-α s^

ω^(2) ^(s) = α^2^se^-α s^

We note that while ω^(1)^(s) is monotonically decreasing, ω^(2)^(s) increases to a maximum at s = 1/α, then decreases monotonically and asymptotically to zero. We choose the second Erlang-function as our kernel because it is the simplest member of the family with a peak. This embodies the concept that, in order to produce its metabolic effect, insulin has to reach the tissues and activate intracellular enzymatic mechanisms (hence its maximal action on glucose metabolism is delayed) and that natural breakdown of insulin induces a progressive loss of effect of increased concentrations of the hormone as they become more distant in the past. A high α value determines a concentrated kernel corresponding to a fast-rising, fast-decaying effect of insulin on peripheral tissues. We therefore set



and we define  as the average time for the metabolic effect of insulin in changing glycemia. The insulin-independent glucose tissue uptake process is modelled as a Hill function rapidly increasing to its (asymptotic) maximum value T_xg_; thus for glycemia values near 2 mM the insulin-independent glucose tissue uptake is already close to its maximum. This formulation is intended to represent the aggregated apparent zero-order (fixed) glucose utilization mechanism at rest (mainly the brain and heart [11]; W. Sacks in [12] p. 320), with the mathematical and physiological requirement that glucose uptake tends to zero as glucose concentration in plasma approaches zero.

The variation of insulin concentration in its distribution space (equation 2) may be thought of as due to the external insulin infusion, glucose dependent pancreatic insulin secretion and the apparently first-order insulin removal from plasma.

We use steady-state conditions to decrease the number of free parameters to be estimated: at steady state, before the start of the clamp (G = G_b_, I = I_b_, T_gx _= T_ix _= 0), we have



Therefore the parameters T_ghb_, T_xg_, and T_iG _are completely determined by the values of the other parameters (and ρ is determined from *α*).

### Statistical analysis

The system (1), (2) and (3) has been numerically integrated by means of a fourth order Runge-Kutta scheme; the solutions thus obtained have been fitted by Weighted Least Squares (WLS) separately on each subject's glycemia and insulinemia time-points, estimating only the free parameters G_b_, I_b_, K_xgI_, K_xi_, T_ghmax_, V_g_, V_i_, *α*, τ_g_, λ. The statistical weight associated with each observed glucose and insulin concentration point has been defined as 1/CV^2^, where CV is the coefficient of variation, equal to 0.015 for glucose and 0.07 for insulin [13]. The weighted quadratic loss function was minimized by a Nelder-Mead simplex algorithm in order to obtain the WLS parameter estimates for each subject. In order to highlight possible physiological differences among subjects depending on their BMI, two groups were defined: a group consisting of lean subjects (BMI ≤ 25) and a group consisting of overweight or obese subjects (BMI > 25). Comparisons of anthropometric characteristics, metabolic characteristics and model parameter values between these groups were performed by the Mann-Whitney U-test owing to the small number of subjects in each group. Comparisons within groups were performed by the Wilcoxon test for matched pairs.

## Results

Table 1 shows anthropometric characteristics (BMI, BSA), measured plasma glucose and insulin concentrations (G_fast_, I_fast_) in the two groups immediately before the clamp, and the average levels of insulin after 80' of clamp insulinization (I_max_). All differences in the characteristics were highly significant, with the median values in the obese/overweight group markedly higher than those in the lean group. Even though there was a significant difference in fasting glycemia between the groups, average levels remained within the norm. However, fasting insulinemia was more than four-fold higher in the obese/overweight group, consistent with what is usually observed in this patient population.

For each parameter fitted and determined, the median, minimum and maximum from the sample of values obtained are reported in Table 4.

**Table 4:** Estimated and determined parameter values for lean (BMI ≤ 25) and overweight or obese (BMI > 25) subjects.

	**Lean (n = 7)**	**Overweight or Obese (n = 8)**	**p**
**Estimated Parameters**			
G_b _[mM]	3.67 [2.80, 4.36]	5.11 [4.52, 5.97]	**0.001**
I_b _[pM]	17.91 [8.59, 63.41]	121.05 [61.55, 256.41]	**0.002**
K_xgI _[min^-1^/pM]	9.94 · [7.1, 21.2] · 10^-6^	6.34 · [0, 13.3] · 10^-6^	0.132
K_xi _[min^-1^]	0.039 [0.022, 0.057]	0.029 [0.021, 0.045]	0.203
T_ghmax _[mmol/min/kgBW]	0.069 [0.05, 0.12]	0.128 [0.026, 0.274]	0.105
V_g _[L/kgBW]	0.49 [0.33, 0.90]	0.47 [0.25, 0.67]	0.643
V_i _[L/kgBW]	0.4 [0.36, 0.78]	0.39 [0.21, 0.65]	0.487
*α *[#]	0.017 [0.015, 0.082]	0.024 [0.008, 0.048]	0.908
τ_g _[min]	3.00 [1.00, 11.50]	5.14 [0.50, 9.00]	0.917
λ [mM^-1^pM^-1^]	8.9 [1.2, 21.3] · 10^-3^	3.1 [0.2, 4] · 10^-3^	**0.037**
**Determined Parameters**			
T_ghb _[mmol/min/kgBW]	0.042 [0.028, 0.052]	0.019 [0.009, 0.117]	0.36
T_xg _[mM / min]	0.085 [0.057, 0.126]	0.046 [0.012, 0.397]	0.203
T_iG _[pM/min/mM]	0.096 [0.031, 0.29]	0.267 [0.128, 0.668]	**0.011**
ρ [#]	115.4 [24.3, 136.2]	83.6 [42.1, 267.7]	0.908

Comparisons were performed by the Mann-Whitney U-test. Values are expressed as median [min, max].

The predicted basal glycemia and insulinemia values (G_b_, I_b_) were close to the observed fasting values and were significantly different between groups (respectively p = 0.001 and p = 0.002). Lean subjects have a greater ability (about 3-fold higher) to reduce hepatic glucose output when glycemia and insulinemia increase (expressed by the parameter λ, p = 0.037). The parameter T_iG _(glucose-dependent pancreatic secretion of insulin) is also significantly different between groups (p = 0.011) and the insulin synthesis rate in obese/overweight subjects is about three-fold higher than in lean subjects. The delay coefficient τ_g _is of the order of 3 to 5 minutes, which seems a reasonable time for glucose infused through an arm vein to be distributed throughout the body, equilibrate, and be detected by sampling through the arterialized contralateral arm vein.

In Table 5 the measured values of the M/I index over the time periods 80'–120' and 260'–300' are shown for normal and obese/overweight subjects: as expected, the rate of glucose uptake per unit plasma insulin concentration is significantly higher in lean subjects in both the 80'–120' (p = 0.001) and the 260'–300' periods (p = 0.015). However, whereas in lean subjects the M/I value remains stable between the two periods (p = 0.6), in the obese/overweight group it increases significantly (p = 0.02).

**Table 5:** M/I index values for lean and overweight or obese subjects measured over the 80'–120' and on the 260'–300' time periods.

	**Lean (n = 7)**	**Overweight or Obese (n = 8)**	**p (M-W U)**
**M / I (80'–120')**	9.75 · 10^-5 ^[6.97, 11.42] · 10^-5^	2.66 · 10^-5 ^[1.57, 5.2] · 10^-5^	**0.001**
**M / I (260'–300')**	8.9 · 10^-5 ^[4.9, 13.2] · 10^-5^	3.86 · 10^-5 ^[2.54, 7.44] · 10^-5^	**0.015**
**p (Wilcoxon)**	0.6	**0.02**	

Comparisons between groups were performed by the Mann-Whitney U-test. Comparisons within groups were performed via the Wilcoxon test for matched pairs. Values are expressed as median [min, max].

**Figure 2:**
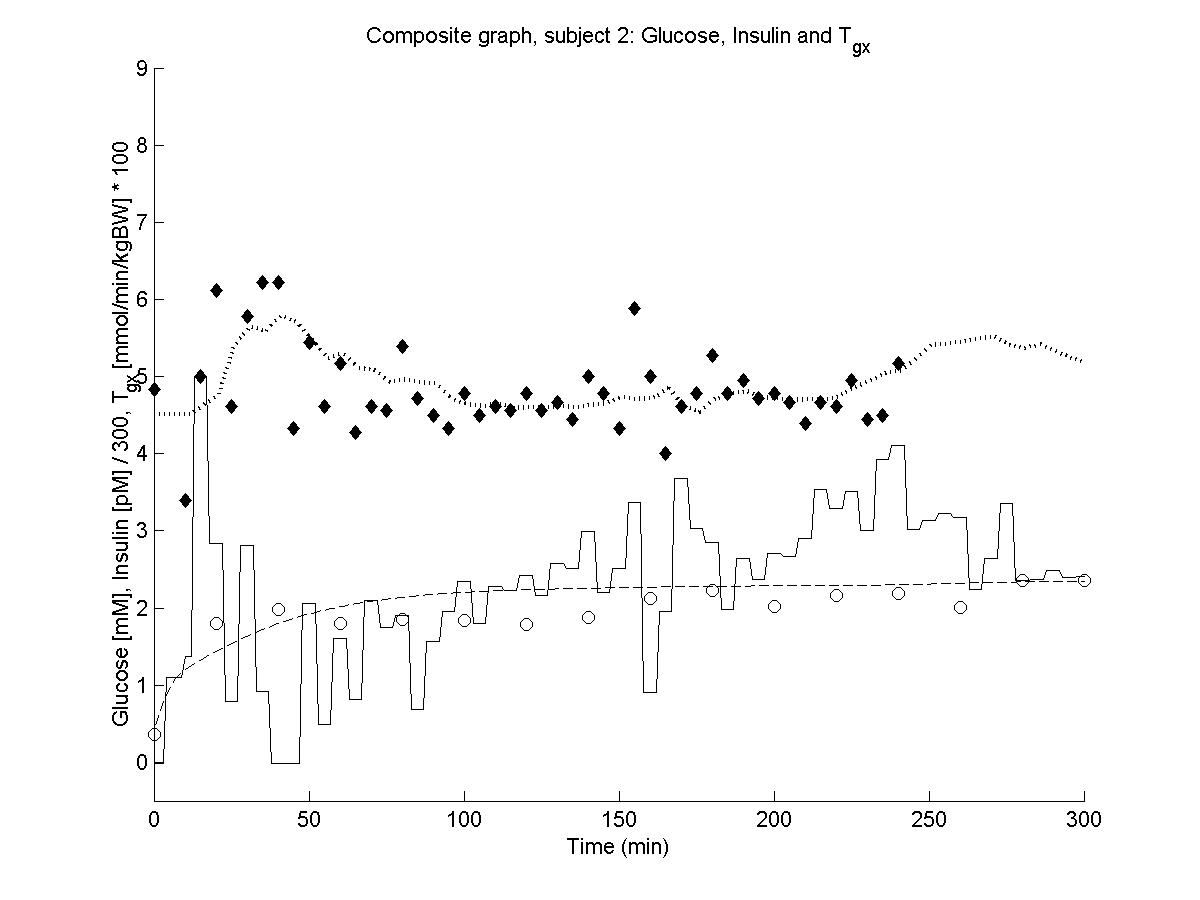
**Composite plot for subject 2 (BMI = 35.9). **Observed (◆) and predicted (....) glycemia; observed (o) and predicted (----) insulinemia; glucose infusion rate (solid line). For ease of comparison, the insulin concentrations and the glucose infusion rates are divided by factors of 300 and 0.01 respectively.

**Figure 3:**
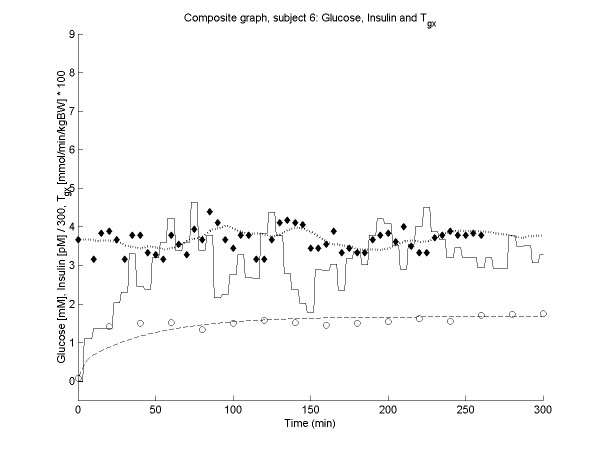
**Composite plot for subject 6 (BMI = 19.33). **Observed (◆) and predicted (....) glycemia; observed (o) and predicted (----) insulinemia; glucose infusion rate (solid line). For ease of comparison, the insulin concentrations and the glucose infusion rates are divided by factors of 300 and 0.01 respectively.

**Figure 4:**
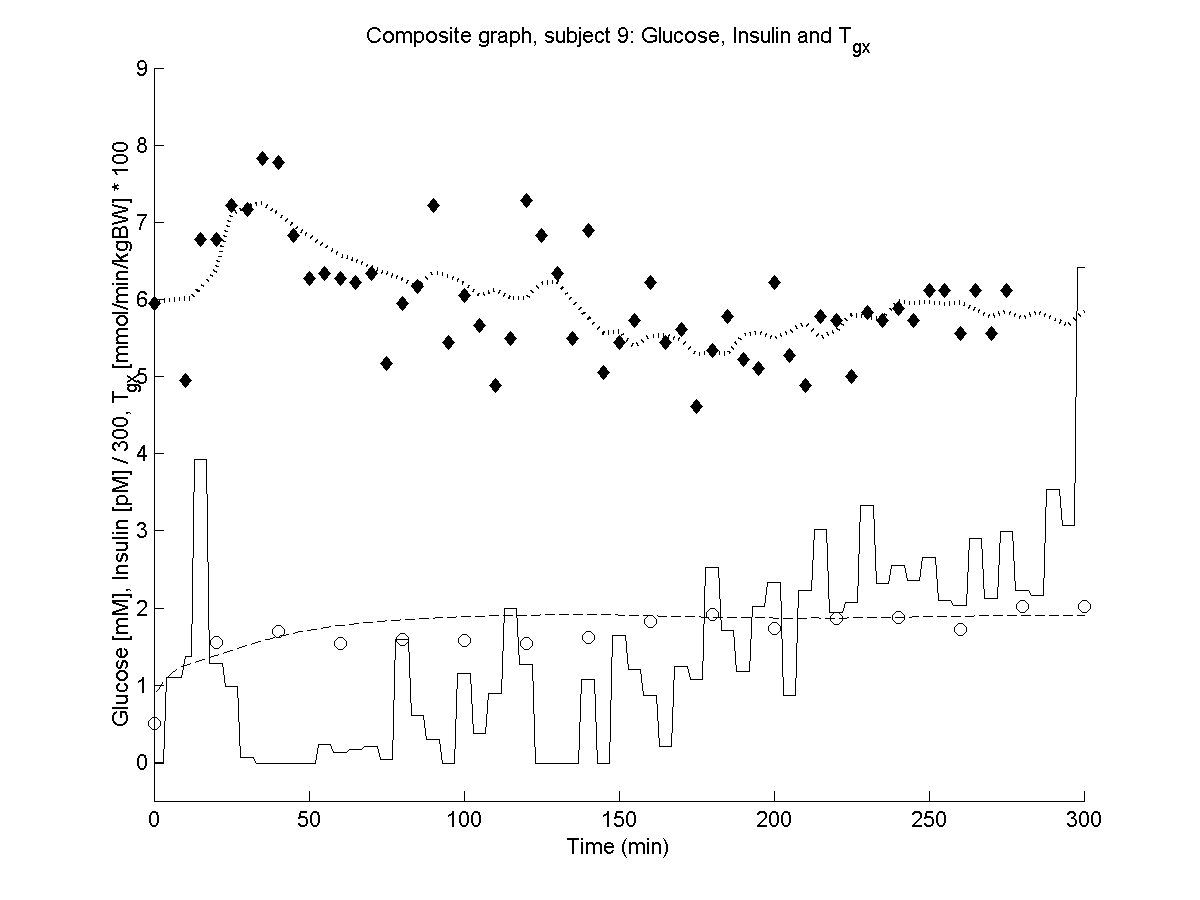
**Composite plot for subject 9 (BMI = 63.6). **Observed (◆) and predicted (....) glycemia; observed (o) and predicted (----) insulinemia; glucose infusion rate (solid line). For ease of comparison, the insulin concentrations and the glucose infusion rates are divided by factors of 300 and 0.01 respectively.

**Figure 5:**
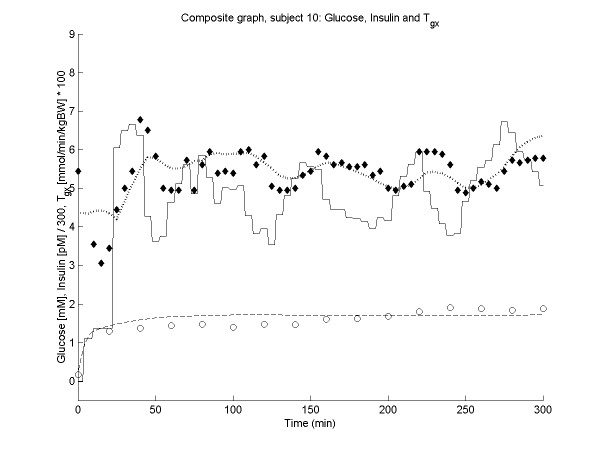
**Composite plot for subject 10 (BMI = 18.6). **Observed (◆) and predicted (....) glycemia; observed (o) and predicted (----) insulinemia; glucose infusion rate (solid line). For ease of comparison, the insulin concentrations and the glucose infusion rates are divided by factors of 300 and 0.01 respectively.

Figures 2, 3, 4, 5 show the time course of observed and predicted glycemia, observed and predicted insulinemia and glucose infusion rate for four experimental subjects (two lean and two obese).

## Discussion

It was shown in the early '80s [7] that a significant increase of glucose tissue uptake during the euglycemic hyperinsulinemic clamp could be obtained in obese subjects by waiting for up to 4–6 hours. This basic observation, confirmed by the series of obese subjects studied in the present work, challenges the assumption that steady state is attained after 2 hours of the clamp, at least in one patient subpopulation of great metabolic interest. Nolan et al. [14], while performing an isoglycemic hyperinsulinemic clamp, also demonstrated a marked delay in activation of whole-body glucose disposal rate, arterio-venous glucose difference and leg glucose uptake in seven subjects with Type 2 Diabetes Mellitus and in seven obese non-diabetic subjects, as compared to healthy controls.

The concept of insulin resistance as a decreased effect of the hormone on whole body glucose uptake can be made more specific: on the one hand we might wish to measure the speed with which a given level of metabolic response is attained; on the other, we might wish to quantify the maximal response attainable by a suitably raised insulin plasma concentration. It is clear now that when using the classical two-hour clamp, subpopulations of subjects respond within different time frames. Concentrating on the level of response at 2 hours would label subjects with a residual metabolic capacity as insulin-resistant: this may or may not be appropriate depending on the mode of insulin resistance that the physiologist is interested in, whether the speed or the capacity of response. The case of the obese subject represents this ambiguity very well: if by insulin resistance we mean the result of the EHC at 2 hours, that is to say a decreased effect of insulin on whole body glucose uptake under hyperinsulinization with respect to a specific and short time frame, then obese subjects can be adequately diagnosed by the clamp as being generally insulin resistant. If, on the other hand, we abandon the time frame requirement and address the maximal ability to respond to the hormone, then the standard clamp procedure is not adequate since it fails to allow slowly-responding subjects to develop a complete response. A way out of this ambiguity for diagnostic purposes could be to use the parameters of a mathematical model of the metabolic response during the clamp. Hopefully, this model would be able to quantify both the maximal response obtainable by the subject and the rate at which this response is generated. Hence the diabetologist would be offered separate, independent and complementary items of information on which to base the diagnosis.

Given the above considerations, the approach followed in the present work was therefore to construct a deterministic mathematical model of the time course of glucose uptake rate during a clamp experiment.

A series of studies [15-17] demonstrated that insulin-stimulated glucose uptake correlates with the appearance of insulin in lymph fluid, a marker for interstitial insulin, rather than with the appearance of insulin in the circulatory stream. Whether trans-endothelial passage of insulin from the circulation to the interstitial space is the sole or the main mechanism for the delay is debatable, even though it may be rate-limiting in the activation of glucose uptake, since the pancreatic response to glucose should be fast and since, once insulin is in the interstitial space, further endocellular steps are very rapid. In any case, out of the many models we tried in order to explain the observed insulin and glucose concentration time courses, the model that best explains the data includes a delay in the action of plasma insulin in correcting glycemia. Of the many alternative explicit representations of such delay that could have been used, one of the simplest was chosen, a Erlang-function kernel, to simplify the model's mathematical treatment.

It has been shown [14] that Hepatic Glucose Output (HGO) suppression after step insulinization is not immediate, HGO decreasing towards 0 in an approximately exponential manner from its pre-insulinization level. In the present work, HGO was not independently measured by tracer techniques. The model proposed here assumes that the variable representing HGO (identified with the symbol T_gh_) falls progressively to a new equilibrium value as delayed insulin increases progressively to its new equilibrium level after a step increase in plasma insulin. In this, our model agrees with Nolan's observation. Further, in the model proposed in the present work, equilibrium T_gh _falls exponentially (with parameter λ) as equilibrium insulin increases from baseline to full insulinization levels.

The two parameters T_ghmax _and K_xgI _express respectively the maximum Hepatic Glucose Output and the sensitivity of glucose uptake to insulin concentration. Neither was significantly different between lean and obese subjects. However, T_ghmax _was higher and K_xgI _was lower in obese subjects, and both these changes would point to a decreased insulin sensitivity in this patient group. While the observed lack of significance may well be a consequence of the limited power of the present study, given the small number of subjects considered, the fact that these two parameters were not much changed in obese subjects while λ was significantly lower again indicates a relative slowness in mounting an appropriate response rather than a relative incapacity to mount a sustained response eventually.

From the modelling point of view, the present study prompts two considerations. The first is that a clamp that is medically very successful (i.e. during which the physician manages to clamp glycemia effectively to within a narrow range) may be less informative about the actual subject's compensation mechanisms than a clamp where imprecise correction of glycemia gives rise to oscillations. The second is that, especially for subjects such as the one reported in Figure 5, where sustained oscillations are produced, random perturbations of the system may give rise to accidental phase shifts. This makes it very hard or impossible to follow the oscillations unless for the model can accommodate random variations of metabolism. Future efforts in modelling the clamp will have to consider this feature.

## Conclusion

In conclusion, the present paper describes a possible deterministic modelling of the EHC, which may prove useful for studying obese subjects who show delayed expression of their maximal increase of glucose uptake under insulinization. Considering the amplitude of response independently of the time factor, the whole body capacity of glucose uptake in obese subjects does not appear to be decreased with respect to lean subjects.

## Competing interests

The author(s) declare that they have no competing interests.

## Authors' contributions

UP: mathematical modeling, statistical analysis, drafting of the manuscript;

ADG: mathematical modeling, drafting of the manuscript;

SP: mathematical modeling;

SD: mathematical modeling;

GM: design of the experiment, collection of data, drafting of the "Experimental protocol" and "Discussion" sections of the manuscript.

All authors read and approved the final manuscript.
